# Classification Accuracy of a Wearable Activity Tracker for Assessing Sedentary Behavior and Physical Activity in 3–5-Year-Old Children

**DOI:** 10.3390/ijerph15040594

**Published:** 2018-03-26

**Authors:** Wonwoo Byun, Jung-Min Lee, Youngwon Kim, Timothy A. Brusseau

**Affiliations:** 1 Department of Health, Kinesiology, and Recreation, University of Utah, Salt Lake City, UT 84112, USA; Youngwon.Kim@mrc-epid.cam.ac.uk (Y.K.); tim.brusseau@utah.edu (T.A.B.); 2 School of Physical Education, Kyung Hee University, Yongin 17104, Korea; jungminlee@khu.ac.kr; 3 Medical Research Council Epidemiology Unit, University of Cambridge School of Clinical Medicine, Cambridge CBD 0SP, UK

**Keywords:** accelerometer, sedentary behavior, physical activity, preschool, children, obesity

## Abstract

This study examined the accuracy of the Fitbit activity tracker (FF) for quantifying sedentary behavior (SB) and varying intensities of physical activity (PA) in 3–5-year-old children. Twenty-eight healthy preschool-aged children (Girls: 46%, Mean age: 4.8 ± 1.0 years) wore the FF and were directly observed while performing a set of various unstructured and structured free-living activities from sedentary to vigorous intensity. The classification accuracy of the FF for measuring SB, light PA (LPA), moderate-to-vigorous PA (MVPA), and total PA (TPA) was examined calculating Pearson correlation coefficients (r), mean absolute percent error (MAPE), Cohen’s kappa (*k*), sensitivity (Se), specificity (Sp), and area under the receiver operating curve (ROC-AUC). The classification accuracies of the FF (ROC-AUC) were 0.92, 0.63, 0.77 and 0.92 for SB, LPA, MVPA and TPA, respectively. Similarly, values of kappa, Se, Sp and percentage of correct classification were consistently high for SB and TPA, but low for LPA and MVPA. The FF demonstrated excellent classification accuracy for assessing SB and TPA, but lower accuracy for classifying LPA and MVPA. Our findings suggest that the FF should be considered as a valid instrument for assessing time spent sedentary and overall physical activity in preschool-aged children.

## 1. Introduction

Accurate monitoring of children’s sedentary behavior (SB) and physical activity (PA) is an integral element of public health as the lack of PA and excessive time spent sedentary are two major contributors to the childhood obesity epidemic worldwide [1,2]. For younger children aged under 6 years old, assessing SB and PA requires careful considerations in the selection of a measurement method due to the intermittent nature of children’s activity and limited ability to recall their habitual activities [3]. In this regard, accelerometry-based activity monitors (accelerometers hereafter) have become the method of choice for measuring SB and PA in young children [4] because accelerometers have several advantages over subjective instruments (i.e., parent proxy-report) such as being free of recall bias and social desirability [3,5].

Despite significant improvements in feasibility, the relatively high cost and complexity of data processing procedures preclude the widespread application of research-grade accelerometers in large-scale research and practical settings [6,7]. Recent advances in wearable sensor technology have offered the abundant availability of activity trackers to researchers and practitioners for quantifying objective data of SB and PA in diverse settings [8,9,10], indicating that the usability of the wearable activity trackers has been well accepted in various population subgroups. 

Among a variety of wearable activity trackers, the Fitbit Flex (FF) has been the best-selling device on the consumer market because it is considered to be the most affordable and versatile model manufactured by Fitbit [11], which has been dominating the global market of wearables [12]. In fact, the FF has been increasingly used in clinical and epidemiological research aimed at promoting PA; recent randomized controlled trials using self-monitoring of PA as a key intervention component concluded that the FF was an effective tool for increasing PA and reducing SB in both general and clinical populations [13,14,15,16].

Owing to the fast-growing popularity and applications of the FF, recent studies have evaluated its accuracy, reporting that the FF provided accurate estimates of SB, PA, and energy expenditure in adults. These studies demonstrated strong correlations (range: *r* = 0.84–0.90), a high classification accuracy (positive predictive value = 99%), and a relatively small measurement error (mean absolute percent error ≈ 15%) of the FF against criterion measures, such as an ActiGraph GT3X+ (ActiGraph Corp., Pensacola, FL, USA) accelerometer, direct and indirect calorimetry, and doubly labeled water [17,18,19]. For young children, however, little is known about the accuracy of the FF activity tracker in quantifying the amount of time spent in SB and PA. Therefore, the purpose of this study was to examine the validity of the FF activity tracker for quantifying SB and varying intensities of PA in preschool-aged children under simulated free-living conditions. 

## 2. Materials and Methods

### 2.1. Participants and Instrument

Twenty-eight healthy 3–5-year-old children (Girls: 46%, Age: 4.8 ± 1.0 years, BMI: 16.4 ± 1.6 kg·m^2^) were recruited from adjacent communities of Fargo, ND and Moorhead, MN, USA. Children who were unable to engage in habitual PA as recommended by their pediatricians or who were physically disabled were excluded from this study. The study protocol was approved by the University Institutional Review Board. The FF (Fitbit Flex 1, Fitbit Inc., San Francisco, CA, USA) is a light (14 g) and small (3.2 × 1.2 × 1.0 cm) device designed to be worn on the wrist and capture bodily movements at vertical, lateral, and anteroposterior planes using a tri-axial accelerometer. Using its proprietary algorithm, the FF transforms raw data into activity counts in 60-s sampling intervals that define activity intensities as 0 = sedentary, 1 = light PA, 2 = moderate PA, 3 = vigorous PA. Directly observed data was used to score intensities of activities children performed during the protocol, and categorized intensities as the same as the FF’s activity count (0–3 scale for sedentary-to-vigorous PA; 0 = sedentary, 1 = light PA, 2 = moderate PA, 3 = vigorous PA); we purposely chose this approach and coded the criterion data in this fashion rather than a particular systematic direct observation system for preschool-aged children, due to a discrepancy in sampling intervals and latencies between the FF and systematic direct observation systems (e.g., FF: 60 s vs. Direct Observation: 5–15 s) [20,21]. In order to make direct comparisons between the FF and direct observation at the same level of sampling interval, minute-by-minute activity counts from the FF were temporally matched with directly observed activity intensity codes, according to prescribed criterion activity intensities as defined according to the Youth Compendium of Energy Expenditure [22].

### 2.2. Procedures

Each participating child, accompanied by his/her parent, reported to the lab on their scheduled day of an experiment and reviewed details on the study protocol. Prior to data collection, written informed consents were obtained from parents/legal guardians of participating children. A parent/guardian reported the child’s demographic information (i.e., date of birth, sex, race/ethnicity) in a short survey. Next, after the child removed shoes and outer clothing, standing height was measured to the nearest 0.1 cm with a pediatric stadiometer (Shorr Productions; Olney, MD, USA), and body weight (to nearest 0.1 kg) was measured using a scale (Seca, Model 770; Hamburg, Germany). To ensure reliability, height and weight were measured twice; if the first two measurements differed by more than 1 cm or 1 kg, respectively, another measurement was taken. Body mass index (BMI) was calculated as weight (kg)/height^2^ (kg/m^2^). BMI-for-age percentile was calculated based on the population mean BMI values reported in the CDC growth charts [23].

The FF device was attached to the child’s non-dominant wrist using an adjustable pediatric wristband. Following this, each participant performed a set of various unstructured and structured free-living activities at sedentary, light, moderate, and vigorous intensity in the laboratory (Table 1). Authors carefully selected all activities performed by children according to the following criteria: (1) age-appropriate activities for preschool-aged children, (2) popular activities in free-living settings (e.g., childcare and home), (3) activities for which children need minimal instructions, and (4) the criterion intensities defined in the Youth Compendium of Physical Activities [22]. Two researchers directly observed and assisted children while performing all activities in order to ensure children engage in all activities with appropriate efforts; the researchers provided positive verbal feedback to encourage the children to complete all the activities at prescribed intensities. Each activity bout lasted 5 min, except for the sedentary activities, which were 8 min long (4 min in supine and 4 min in a position). During the transition periods between each activity bout, the child received incentives such as stickers and small toys, and compliments from research staff and parents. The total duration of the whole activity protocol was 34 min, including 1-min transition periods. All activity protocols progressed from sedentary activities to vigorous intensity following Welk’s recommendations [24]. 

### 2.3. Statistical Analyses

Univariate statistics (Mean, SD, and percentile) were calculated to summarize descriptive characteristics of participants, and independent t-tests were used to examine sex differences in those descriptive characteristics. Overall agreement and classification accuracy of the FF against direct observation were evaluated using the following statistical analyses: (1) Spearman’s rank order correlation coefficients (ρ) examining the relative agreement in minutes of each intensity activity between the FF and direct observation (0–3 scale for sedentary-to-vigorous PA); (2) mean absolute percent error (MAPE) examining the measurement errors of the FF; (3) Cohen’s kappa evaluating the levels of agreement on activity intensity classification between the FF and direct observation, and (4) sensitivity (Se), specificity (Sp), and area under the receiver operating curve (ROC-AUC) to determine the classification accuracy of the FF. All statistical analyses were performed using STATA Version 14 (StataCorp, College Station, TX, USA), and statistical significance was set at α = 0.05.

### 2.4. Ethical Statement

All subjects gave their informed consent for inclusion before they participated in the study. The study was conducted in accordance with the Declaration of Helsinki, and the protocol was approved by the Ethics Committee of North Dakota State University (IRB Approval of Protocol number HE15188).

## 3. Results

Descriptive characteristics of children who participated in this study are presented in Table 2; no significant differences were observed between boys and girls across demographic and anthropometric characteristics. 

When compared with direct observation, on average, the FF recorded higher minutes of SB (absolute mean difference (AMD) = 2.3 min) and light PA (LPA; AMD = 4.6 min), but lower minutes of moderate-to-vigorous PA (MVPA; AMD = 6.9 min) and total PA (TPA; AMD = 2.3 min) (Table 3). MAPEs were lower for SB (MAPE: 28.8%) and TPA (MAPE: 11.5%) than for MVPA (MAPE: 46%) and LPA (MAPE: 92%). As also shown in Table 3, correlations between direct observation and the FF were statistically significant and high for SB, TPA and MVPA, but considerably low and insignificant for LPA. 

Results from 2 × 2 contingency tables, kappa coefficients, area under the receiver operating curve (ROC-AUC) analyses, and sensitivity (Se) and specificity (Sp) calculations are shown in Table 4. Levels of agreement between the FF and direct observation determined by kappa coefficients were moderate for SB (*k* = 0.78) and TPA (*k* = 0.78), but weak for LPA (*k* = 0.18) and MVPA (*k* = 0.51). Across activity intensities, all calculated values of Se, Sp, and percentage of correct classification were higher for SB and TPA than for LPA and MVPA, with an exception of the highest Sp for MVPA. Based on the standard for ROC-AUC value interpretation (excellent: 0.9–1.0, good: 0.8–0.9, fair: 0.7–0.8, poor: <0.7) [25], the classification accuracy of the FF was excellent for SED (ROC-AUC: 0.92) and TPA (ROC-AUC: 0.92), fair for MVPA (ROC-AUC: 0.77), and poor for LPA (ROC-AUC: 0.63). 

## 4. Discussion

To our knowledge, this is the first study to determine whether a wearable activity tracker can accurately classify SB and PA intensity in preschool-aged children. We found that the FF classified SB and TPA accurately but was inaccurate for classifying LPA and MVPA in 3 to 5-year-old children. Given the need for more feasible options for measuring preschoolers’ SB and PA, our findings provide important implications for future surveillance and interventions that intend to incorporate objective assessment of SB and PA in preschoolers. More specifically, using the FF children’s time spent in SB and overall PA can be easily tracked in real-time without retrieving the device and downloading the data, which would enhance data collection and management procedures. Thus, it is likely to facilitate the implementation of large-scale surveillance and the development of cost-effective interventions targeting the promotion of PA in preschool-aged children. In support, a recent review reported that a Fitbit device was the most widely used wearable activity tracker in biomedical research, accounting for 89% of published research utilizing wearables, and 83% of clinical trials and 95% of NIH-funded research using wearables [26].

Findings from this study are important not only for researchers, but also for practitioners in early childhood education and public health settings. Several governmental public health agencies have provided PA guidelines specific to preschool-aged children, which recommend ≥3 h/day of TPA [27,28,29] or ≥15 min/h of TPA while children are attending childcare centers or preschools [30]. As part of tracking progress towards promoting PA both in and outside of childcare settings, practitioners such as preschool teachers, early childhood education agents, and public health agents should evaluate their compliance with these guidelines. In view of the observed high accuracy of the FF for measuring TPA and its ease of use, practitioners can consider the FF a feasible device for monitoring PA in childcare settings. 

It is noteworthy to compare the classification accuracy of the FF found in this study with that of research-grade accelerometers. Janssen et al. evaluated the classification accuracy of the ActiGraph GT3X accelerometer in preschool-aged children using direct observation as a criterion during 150 min of semi-structured activity protocol [31]; the authors reported that classification accuracy of the GT3X with varying cutpoints was good for SB (ROC-AUC range: 0.80–0.64), poor for LPA (ROC-AUC range: 0.50–0.65), and fair for MVPA (ROC-AUC range: 0.62–0.72). This indicates that the classification accuracy of the ActiGraph GT3X monitor was relatively low for LPA and MVPA, which is in line with findings from the present study. 

Another recent study that evaluated the agreement between the GT3X accelerometer and direct observation in a small sample of 4 and 5-year-old children (*N* = 12) during unstructured outdoor free-play showed consistent findings with our results in terms of the pattern that the levels of agreement (i.e., kappa, Se, Sp, % correctly classified) were higher for SB than for MVPA [32]. Moreover, the degree of measurement agreement for the ActiGraph accelerometer found in the previous study [32] was not superior to that which the FF observed in the present investigation. This suggests that the FF may serve as a viable alternative to the GT3X, the most widely used research-grade accelerometer, in measuring preschoolers’ habitual activity under free-living conditions. However, more research is needed to evaluate the accuracy of both the FF and GT3X altogether in independent samples of preschool-aged children. 

Another important finding from the present study was the observed lower accuracy of the FF in classifying LPA and MVPA. The FF utilizes proprietary algorithms that set its sampling interval at 60-s, but the shorter epochs (5–15-s) have been recommended to better capture sporadic activity patterns and intermittent accumulations of MVPA in young children [7,33]; Reilly et al., reported that preschoolers’ MVPA was underestimated up to 40% using a 60-s epoch when compared to using 15-s epoch [34], indicating that the observed underestimation and low sensitivity of the FF for measuring MVPA in our results could be explained by the influence of the longer epoch used by the FF. In terms of the FF’s low classification accuracy for LPA found in this study, no information about the effect of epoch lengths on estimating LPA in preschool-aged children was available at the moment this study was conducted; however recent studies using the FF reported the greater measurement errors (i.e., MAPE) for LPA (i.e., slow walking) than MVPA in healthy adults [35,36]. Therefore, it is necessary to exercise caution when using the FF for measuring preschoolers’ LPA.

This study has both notable strengths and limitations. A strength of this study was the use of age-appropriate and naturalistic activity protocol rather than strictly structured activities (e.g., treadmill walking and running), which enabled the mimicking of children’s true free-living activities. We also achieved a very high rate (93%) of compliance with the activity protocol by implementing naturalistic strategies for young children (e.g., having research assistant play with children, allowing children to play with their siblings). However, the relatively small sample size and short duration of activity protocol, especially for light intensity, which composes a sizable portion of waking hours in young children, are limitations of this study. Therefore, additional research with a larger sample size and longer duration of activity protocol at varying intensities of activity is recommended to fully determine the classification accuracy of the FF in preschoolers. 

## 5. Conclusions

The FF demonstrated excellent classification accuracy for assessing SB and TPA, but lower accuracy for classifying LPA and MVPA. Considering the observed high accuracy of the FF for classifying SB and TPA in this study, we suggest that the FF should be considered as a valid instrument for assessing time spent sedentary and overall physical activity in preschool-aged children. As technological advances in wearable devices will continuously offer more feasible and valid options for measuring habitual activity in young children, pediatric researchers and practitioners need to be informed on the accuracy of these consumer wearables that have significant potential for research and practical applications. 

## Figures and Tables

**Table 1:** Description of performed activities by intensity.

Intensity	Activities	Duration	METs	Description
**Sedentary**	TV watching—Lying down	4 min	1.2 MET	Children lay in the supine position on a cushioned mat while watching an age-appropriate movie.
	TV watching—Sitting in a couch	4 min	1.4 MET	Children sat in a child-sized chair while watching an age-appropriate movie.
**Light**	Playing with small toys	5 min	1.5–3.0 MET	On a rubber floor, children played with a variety of toys that do not require moderate-to-hard efforts (e.g., building blocks, miniature cars, stuffed animals, and puzzles).
**Moderate**	Exploring at fast walking/self-paced running	5 min	4.6 MET	Children participated in a scavenger hunt in which they quickly walked/ran around the lab to find hidden toys. These activities led to sporadic running and required children’s moderate efforts.
**Vigorous**	Soccer/Running	5 min	≥6.0 MET	Children dribbled and kicked soccer balls into a net, chased after it, and simulated soccer game with the assistants.
	Basketball/Ball games (vigorous)	5 min	≥6.0 MET	Children dribbled, shot, retrieved basketballs using a 4-ft hoop without stopping. Children continuously threw balls against a Tchoukball (throwing) net. The children also chased rebounded balls. These activities required continuous running and jumping without stopping at children’s hard efforts.

MET, metabolic equivalent of task.

**Table 2:** Descriptive characteristics of the participants, mean (standard deviation) or percentile.

Characteristic	All (*N* = 28)	Boys (*N* = 15)	Girls (*N* = 13)	*p*-Value
Age (year)	4.8 (1.0)	4.8 (1.1)	4.9 (0.9)	0.68
Height (cm)	108.6 (9.2)	109.0 (11.5)	108.2 (6.5)	0.53
Weight (kg)	19.3 (3.3)	19.8 (3.9)	19.0 (2.8)	0.36
Body Mass Index (kg/m^2^)	16.4 (1.5)	16.5 (1.5)	16.2 (1.7)	0.70
BMI percentile (%)	66 (27.0)	67.7 (26.2)	64.0 (28.8)	0.49
Waist Circumference (cm)	50.0 (3.7)	50.8 (3.8)	50.0 (3.7)	0.69

**Table 3:** Estimated means (SD), absolute mean difference, mean absolute percent error, and Spearman correlation coefficients between direct observation and the Fitbit Flex.

Activity Intensity	Direct Observation (min)	Fitbit Flex (min)	Absolute Mean Difference (min) ^†^	MAPE (%)	Rho (ρ)
SED	8.0	10.3 (1.8)	2.3	28.8%	0.81 *
LPA	5.0	9.6 (4.5)	4.6	92.0%	0.21
MVPA	15.0	8.1 (4.8)	6.9	46.0%	0.62 *
TPA	20.0	17.7 (1.7)	2.3	11.5%	0.81 *

SED, sedentary; LPA, light physical activity; MVPA, moderate-to-vigorous physical activity; TPA, total physical activity; MAPE, mean absolute percent error ^†^ Absolute Mean Difference = │DO–FF│, * *p* < 0.05.

**Table 4:** Agreements in activity intensity classifications between direct observation and Fitbit Flex, kappa, receiver operating curve (ROC-AUC), sensitivity, and specificity.

	Fitbit Flex	Direct Observation			*k*	ROC-AUC (95% CI)	Sensitivity (%)	Specificity (%)	Correctly Classified (%)
		Yes	No	Total					
**SED**	**Yes**	213	69	282	0.78	0.92 (0.90–0.94)	96.8	88.6	90.2
	**No**	7	491	498					
	**Total**	220	560	780					
**LPA**	**Yes**	77	192	269	0.18	0.63 (0.58–0.67)	55.0	70	67.3
	**No**	63	448	511					
	**Total**	140	640	780					
**MVPA**	**Yes**	224	2	226	0.51	0.77 (0.74–0.79)	53.3	99.4	74.8
	**No**	196	358	554					
	**Total**	420	360	780					
**TPA**	**Yes**	488	7	495	0.78	0.92 (0.90–0.94)	88.6	96.8	90.2
	**No**	72	213	285					
	**Total**	560	220	780					

CI, Confidence Interval; *k*, kappa statistic; ROC, receiver operating curve; AUC, area under curve.
